# Secondary analysis of preoperative predictors for acute postoperative exacerbation in interstitial lung disease

**DOI:** 10.1038/s41598-023-41152-y

**Published:** 2023-08-25

**Authors:** Fumiko Seto, Gaku Kawamura, Keisuke Hosoki, Michiko Ushio, Taisuke Jo, Kanji Uchida

**Affiliations:** 1 Department of Anesthesiology, Faculty of Medicine, Graduate School of Medicine, The University of Tokyo, 7-3-1 Hongo, Bunkyo-ku, Tokyo, 113-8655 Japan; 2 Department of Respiratory Medicine, Graduate School of Medicine, The University of Tokyo, Tokyo, Japan; 3 Department of Health Services Research, Graduate School of Medicine, The University of Tokyo, Tokyo, Japan

**Keywords:** Outcomes research, Risk factors

## Abstract

This study assessed whether perioperative management is associated with postoperative acute exacerbations (AEs) in interstitial lung disease (ILD) patients. Using secondary data from the study “Postoperative acute exacerbation of interstitial lung disease: a case–control study,” we compared the perioperative clinical management of the AE and non-AE groups (1:4 case–control matching) selected by sex, year of surgery (2009–2011, 2012–2014, and 2015–2017), and multiple surgeries within 30 days. We compared 27 and 108 patients with and without AEs, respectively. Rates of one lung ventilation (OLV) cases (70 vs. 29%; OR, 5.9; 95%CI, 2.34–14.88; *p* < 0.001) and intraoperative steroid administration (48 vs. 26%; OR, 2.65; 95%CI, 1.11–6.33; *p* = 0.028), and average mean inspiratory pressure (9.2 [1.8] vs. 8.3 [1.7] cmH_2_O; OR, 1.36; 95%CI, 1.04–1.79; *p* = 0.026), were significantly higher in the AE group. There was a significant difference in OLV between the groups (OR, 4.99; 95%CI, 1.90–13.06; *p* = 0.001). However, the fraction of inspired oxygen  > 0.8 lasting > 1 min (63 vs. 73%, *p* = 0.296) was not significantly different between the groups. OLV was significantly associated with postoperative AEs in patients with ILD undergoing both pulmonary and non-pulmonary surgeries. Thus, preoperative risk considerations are more important in patients who require OLV.

## Introduction

Acute exacerbation (AE) of interstitial lung disease (ILD) is one of the most common postoperative pulmonary complications^1^. The mortality rate due to AEs after pulmonary resection is 33.3–100%^2,3^, and fatal outcomes from AEs have been reported in patients with or without pulmonary resection^4,5^. Therefore, it is essential to identify high-risk patients preoperatively and to manage them without AEs in the perioperative period.

In previous studies, most risk factors for postoperative AEs were related to patient status and type of surgery. In 2014, Sato et al. identified the following predictors of AEs after pulmonary resection for lung cancer: surgical procedures, male sex, history of exacerbation, preoperative steroid use, serum sialylated carbohydrate antigen KL-6 levels, usual interstitial pneumonia appearance on computed tomography (CT), and reduced percentage predicted vital capacity^6^. Recently, Hosoki et al. demonstrated the following predictors of postoperative AEs in ILD after pulmonary and non-pulmonary surgeries: honeycombing on CT, percentage predicted forced vital capacity (FVC) < 80%, and Assess Respiratory Risk in Surgical Patients in Catalonia (ARISCAT) risk score ≥ 45^7^. These factors are preoperative risk factors for postoperative AEs in patients with ILD and are therefore used when considering indications for surgery. Furthermore, these predictors allow anesthesiologists and surgeons to evaluate the possibility of postoperative AEs in patients with ILD before surgery without an experienced respiratory physician. However, Hosoki et al. demonstrated risk factors for patient-related AEs and not the risk of perioperative clinical management. Up to now, perioperative management may affect postoperative AEs, there are few reports on perioperative management associated with AEs in patients with ILD despite it is highly anticipated.

A high fraction of inspired oxygen (F_I_O_2_) causes absorptive atelectasis and an increased generation of reactive oxygen species and proinflammatory cytokines, which can result in lung injury^8–10^. Therefore, empirically, a perioperative ventilatory strategy for patients with ILD is to set the F_I_O_2_ as low as possible. Furuya et al. reported that anesthetic management with propofol may be a risk factor for AEs after non-pulmonary resection surgery in patients with idiopathic interstitial pneumonia (IIP)^5^. Few other reports exist on the risk factors for anesthetic management that are associated with postoperative AEs. However, some reports suggest that the risk of AEs does not significantly differ between laparoscopic and open surgery, although hypercapnia and prolonged operative time are concerns^11^. Although perioperative pain may be a cause of prolonged postoperative recovery, few reports show an association between postoperative analgesia and postoperative AEs. Hickling et al. reported that low tidal volume management is recommended for patients with acute respiratory distress syndrome (ARDS), including hypercapnia^12^. Neto et al. reported that maintaining a driving pressure below a certain level and appropriate intraoperative positive end-expiratory pressure (PEEP) may reduce postoperative respiratory complications^13^. These methods may be adapted to intraoperative ventilation in patients with ILD, but there are few reports on ventilation management in patients with ILD.

There is no clear association between anesthesia management and postoperative AEs in patients with ILD. In this report, we performed a secondary analysis of the retrospective observational study by Hosoki et al. titled “Predictors of postoperative acute exacerbation of interstitial lung disease: a case–control study” to elucidate whether perioperative management is associated with postoperative AEs in patients with ILD.

## Methods

We performed a secondary analysis of the data presented in the publication of Hosoki et al. titled “Predictors of postoperative acute exacerbation of interstitial lung disease: a case–control study.” The study involved patients with ILD who underwent surgery under general anesthesia at the University of Tokyo Hospital between January 2009 and December 2017. Two or more respiratory physicians searched the electronic medical records of patients in the cohort to identify those with pre-existing ILD at the time of operations. A matched 1:4 case–control study was performed among 700 surgical patients with ILD matched by sex, year of surgery (2009–2011, 2012–2014, and 2015–2017), and multiple operations within 30 days. Patients under 18 years of age who had undergone solid organ or bone marrow transplantation were excluded. Furthermore, multiple operations within 30 days were considered as a single operation, and the first operation was adopted. Demographic and other baseline characteristics and the presence of blood transfusion were based on the original data from the previous study, and other data in our study were extracted from the anesthesia database. The anesthesia database contains minute-by-minute records of vital signs, ventilation settings, and medications of patients undergoing surgery. Patients who underwent surgery without mechanical ventilation and those with missing data were excluded. The definition of AE in our study was based on the ATS/ERS/JRS/ALAT statement^14^ similar to the previous study: (1) worsening of dyspnea within one month, (2) evidence of hypoxemia, (3) new radiographic alveolar infiltrates, and (4) the absence of alternative explanations such as infection, pulmonary embolism, pneumothorax, or heart failure.

We conducted a matched case–control study. The cases were patients with ILD who developed AE; matched controls were selected from the remaining cohort of ILD patients who did not develop AE. In our study, we compared anesthetic management factors in the postoperative AE and non-AE groups.

Intraoperative factors were collected for the following parameters: method of anesthesia (volatile or intravenous), presence of one lung ventilation (OLV), steroid use, type of surgery (laparoscopic or open), duration of anesthesia, blood loss, in–out balance, blood transfusion, postoperative analgesia methods (opioid and local anesthesia), and intraoperative desaturation events (pulse oximetry value [SpO_2_] ≤ 90% for > 1 min) or hypercapnia (end-tidal carbon dioxide [EtCO_2_] > 60 mmHg for > 1 min). Data on intraoperative respiratory parameters such as mean inspiratory pressure, mean PEEP, F_I_O_2_, and the duration of mechanical ventilation were also extracted. In this study, mean inspiratory pressure and PEEP were measured during mechanical ventilation. Patients with hyperoxia were defined as having an F_I_O_2_ > 0.8 for > 1 min during mechanical ventilation.

This study was approved by the Research Ethics Committee, Graduate School of Medicine and Faculty of Medicine, The University of Tokyo (IRB# 2203, October 2008 to June 2022). Due to the nature of the retrospective study, informed consent was waived by the Research Ethics Committee, Graduate School of Medicine and Faculty of Medicine, The University of Tokyo, provided that eligible patients were offered the opportunity to opt out. All study protocols were conducted in accordance with the amended Declaration of Helsinki.

### Statistical analysis

Comparisons of demographic and other clinical characteristics between the two groups were made using Student’s t-test for continuous parametric variables and the chi-square test or Fisher’s exact test for categorical variables. Comparisons of intraoperative factors between the two groups were made using simple logistic regression analysis, and the odds ratio and 95%CIs were calculated for each risk factor. We considered *p* < 0.05 as a significant difference. Furthermore, factors that showed significant differences in the univariate analysis were used as independent factors, and multiple logistic regression analysis was performed for risk factors found to be significant. Goodness of fit was tested using the Hosmer–Lemeshow test. The discrimination power of significant factors was estimated using area under the receiver operating characteristic curve (AUC). All statistical analyses were performed using IBM Statistical Package for Social Sciences (SPSS) version 25 (IBM, Armonk, NY, USA).

## Results

**Figure 1:**
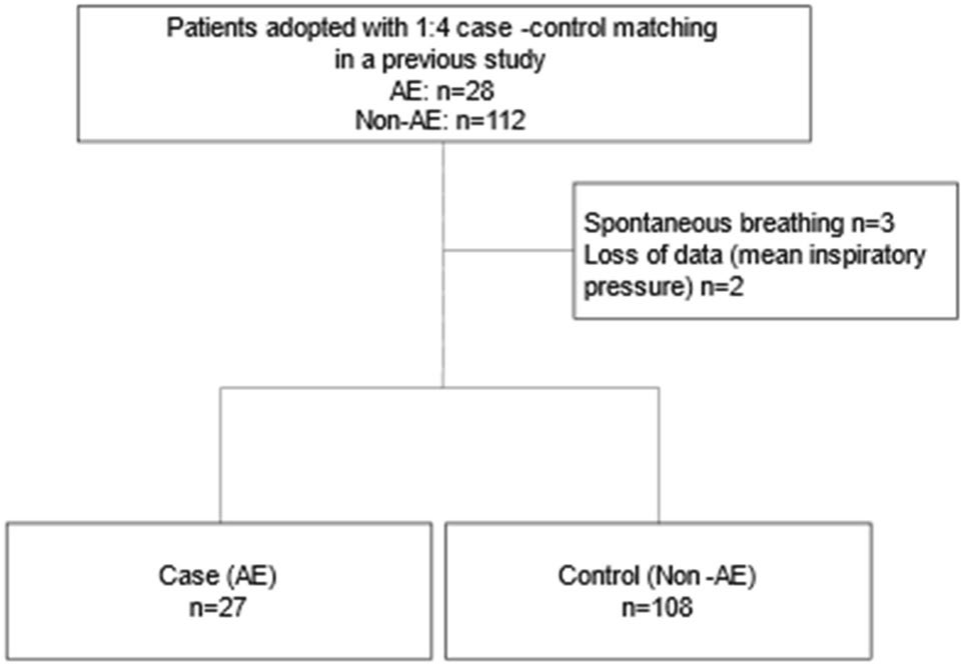
Flow diagram of patient recruitment. AE, acute exacerbation.

Among the 28 cases of AEs and 112 cases of non-AEs in our previous dataset, one case in the AE group and two cases in the non-AE group were excluded because these patients underwent surgery without mechanical ventilation. Two patients in the non-AE group lacked adequate intraoperative data and were therefore excluded (Fig. 1). Therefore, the total number of patients included differed from that in the study by Hosoki et al. However, the patient background was similar in both groups, even after the exclusion of five cases (Table 1). Moreover, 11 patients (41%) in the AE group died during hospitalization. Postoperatively, 22 (81%) and 102 (94%) patients in the AE and non-AE groups, respectively, were extubated in the operating room, whereas five (19%) patients in the AE group and six (5.6%) patients in the non-AE group were transferred to the intensive care unit (ICU) while. Pulmonary surgery performed 15 (56%) and 25 (23%) patients in the AE and non-AE groups, respectively. Five (19%) patients in the AE group and six (6%) patients underwent OLV in non-pulmonary surgery. The surgical site and type of surgery for each case in the AE and non-AE groups are shown in the supplementary Tables S1 and S2, and their percentages are shown in supplementary Figs. S1 and S2.

**Table 1:** Demographic and other clinical characteristics.

Factor	AE n = 27	Non-AE n = 108	*P* value
Age, year, mean (SD)	72.2 (10.7)	68.4 (12)	0.13
Male sex, n (%)	21 (78)	82 (76)	0.84
Height, cm, mean (SD)	159.9 (10.1)	161.8 (7.5)	0.27
Weight, kg, mean (SD)	57.8 (12.1)	59.3 (11.8)	0.55
BMI, kg/m^2^, mean (SD)	22.5 (3.4)	22.5 (3.5)	0.92
ASA-PS, n (%)			0.22
1	2 (7.4)	10 (9)	
2	17 (63)	67 (62)	
3	6 (22)	30 (28)	
4	2 (7.4)	1 (0.9)	
Emergency surgery, n (%)	0 (0)	6 (5.6)	0.26
Surgery duration category, n (%)			0.43
≤ 2 h	6 (22)	34 (31)	
> 2–3 h	4 (15)	21 (19)	
≥ 3 h	17 (63)	53 (49)	
Smoking, n (%)			0.28
Current	3 (11)	21 (19)	
Former	16 (59)	46 (43)	
Never	8 (30)	41 (38)	
Comorbidity, n (%)			
Asthma	1 (3.7)	6 (5.6)	0.58
COPD	7 (26)	18 (17)	0.27
Respiratory infection before surgery	2 (7.4)	7 (6.5)	0.57
Hb, g/dL, mean (SD)	11.6 (2.0)	12.4 (2.0)	0.053
Preoperative steroid use, n (%)	6 (22)	19 (18)	0.58
Reoperation within 1 month, n (%)	5 (19)	24 (22)	0.68

*BMI* body mass index, *ASA-PS* American Society of Anesthesiologists physical status classification, *COPD* chronic obstructive pulmonary disease.

^†^Demographic and other clinical characteristics were compared between cases and controls. Student’s t-test was used for comparisons of continuous variables. The χ^2^ test or Fisher’s exact test was used for comparisons of categorical variables.

### Characteristics of surgery and anesthesia management

There were nine (33%) total intravenous anesthesia (TIVA) cases out of 27 in the AE group and 27 (25%) out of 108 in the non-AE group, and this difference was not significant (*p* = 0.38). The rate of OLV cases was higher in the AE group (19, 70%) compared to that in the non-AE group (31, 29%) (OR, 5.9; 95%CI, 2.34–14.88; *p* < 0.001). There were 13 (48%) patients in the AE group and 28 (26%) patients in the non-AE group who received intraoperative steroid administration. The rate of intraoperative steroid administration was higher in the AE group (OR, 2.65; 95%CI, 1.11–6.33; *p* = 0.028). Two patients (7.4%) in the AE group and 14 (13%) in the non-AE group underwent laparoscopic surgery. The proportion of laparoscopic surgery and duration of anesthesia were not significantly different between the AE and non-AE groups (*p* = 0.43 and *p* = 0.36, respectively). Blood loss and in–out balance did not significantly differ between the groups (*p* = 0.069 and *p* = 0.44, respectively). Seven patients (26%) in the AE group and 18 (17%) in the non-AE group received blood transfusions (*p* = 0.26). There were 12 (44%) cases of perioperative opioid use in the AE group and 56 (52%) in the non-AE group (*p* = 0.49). The number of cases reporting the use of local anesthesia was 14 (52%) in the AE group and 61 (56%) in the non-AE group (*p* = 0.67) (Table 2). Regarding intraoperative events, there were 20 (74%) desaturation cases in the AE group and 60 (56%) in the non-AE group (*p* = 0.09). The number of hypercapnia cases was 5 (19%) in the AE group and 12 (11%) in the non-AE group. No significant differences were observed between the groups (*p* = 0.31).

**Table 2:** Risk factors for surgery and anesthesia management: simple logistic regression analysis.

Factor	AE n = 27	Non-AE n = 108	OR	95%CI	*P* value
Anesthesia method (TIVA), n (%)	9 (33)	27 (25)	1.5	0.60–3.73	0.38
One lung ventilation, n (%)	19 (70)	31 (29)	5.9	2.34–14.88	< 0.000
Intraoperative steroid administration, n (%)	13 (48)	28 (26)	2.65	1.11–6.33	0.028
Laparoscopic surgery, n (%)	2 (7.4)	14 (13)	0.54	0.11–2.52	0.43
Duration of anesthesia, min, mean (SD)	352 (199)	315 (187)	1.0	1.0–1.0	0.36
Blood loss, mL, mean (SD)	955 (2186)	382 (648)	1.0	1.0–1.0	0.069
In–out/weight/time, mL/kg/h, mean (SD)	4.0 (2.4)	4.4 (2.7)	0.94	0.81–1.10	0.44
Transfusion, n (%)	7 (26)	18 (17)	1.77	0.65–4.80	0.26
Opioid use for postoperative pain, n (%)	12 (44)	56 (52)	0.74	0.32–1.73	0.49
Local anesthesia use for postoperative pain, n (%)	14 (52)	61 (56)	0.83	0.36–1.93	0.67
SpO_2_ ≤ 90%, n (%)	20 (74)	60 (56)	2.29	0.89–5.86	0.09
EtCO_2_ > 60 mmHg, n (%)	5 (19)	12 (11)	1.82	0.58–5.69	0.31

TIVA, Total intravenous anesthesia; SpO_2_, Saturation of percutaneous oxygen; EtCO_2_, End tidal carbon dioxide.

### Intraoperative ventilation management

The mean airway pressure during mechanical ventilation was 0.9 cmH_2_O higher in the AE group than in the non-AE group (9.2 ± 1.8 vs. 8.3 ± 1.7 cmH_2_O; OR, 1.36; 95%CI, 1.04–1.79; *p* = 0.026). No significant differences were observed in the mean PEEP between the groups (*p* = 0.47). Seventeen cases (63%) in the AE group and 79 cases (73%) in the non-AE group had intraoperative hyperoxic exposure (i.e., F_I_O_2_ > 0.8 for > 1 min) (*p* = 0.30). Mean inspiratory oxygen during mechanical ventilation did not significantly differ between the groups (*p* = 0.31). The duration of mechanical ventilation was not statistically different between the groups (*p* = 0.37) (Table 3).

**Table 3:** Risk factors for intraoperative ventilation management: simple logistic regression analysis.

Factor	AE n = 27	Non-AE n = 108	OR	95%CI	*P* value
Mean airway pressure during mechanical ventilation, cmH_2_O, mean (SD)	9.2 (1.8)	8.3 (1.7)	1.36	1.04–1.79	0.026
PEEP, cmH_2_O, mean (SD)	3.8 (1.3)	4.0 (1.4)	0.89	0.66–1.21	0.47
Incidence of hyperoxic exposure (F_I_O_2_ > 0.8 for > 1 min), n (%)	17 (63)	79 (73)	0.62	0.26–1.52	0.30
Mean inspiratory oxygen, %, mean (SD)	51.5 (16.4)	48.2 (14.8)	1.01	0.99–1.04	0.31
Duration of mechanical ventilation, min, mean (SD)	334 (200)	298 (186)	1.0	1.0–1.0	0.37

PEEP, Positive end-expiratory pressure; F_I_O_2_, Fraction of inspired oxygen.

### Multiple logistic regression analysis of potential risk factors

We performed multiple logistic regression to identify potential risk factors, such as OLV, intraoperative steroid administration, and the average mean inspiratory pressure during intubation. The results revealed a significant difference in OLV (OR, 4.99; 95%CI, 1.91–13.06; *p* = 0.001) (Table 4).

**Table 4:** Results of multiple logistic regression analysis of potential risk factors.

Factor	OR	95%CI	*P* value
One lung ventilation	4.99	1.91–13.06	0.001
Intraoperative steroid administration	2.42	0.95–6.18	0.065
Mean airway pressure during mechanical ventilation, cmH_2_O	1.19	0.90–1.58	0.23

*p* = 0.233 obtained by Hosmer–Lemeshow test, area under the curve (AUC) = 0.756.

## Discussion

In this case-matched retrospective observational study, we found that OLV, intraoperative steroid administration, and high mean inspiratory pressure were potential predictors of postoperative AEs during general anesthesia in pulmonary and non-pulmonary surgeries. On the contrary, high intraoperative F_I_O_2_ levels were not revealed to be associated with postoperative AEs. Multiple logistic regression indicated a significant association between OLV and postoperative AEs.

The AEs occurred in patients who underwent OLV, even in non-pulmonary surgery. It has been suggested that OLV itself is a risk factor for the development of AEs. In addition to pulmonary surgery (14 cases), this study included one case of distal arch descending aorta replacement, one case of thoracoscopic thymectomy, and three cases of esophagectomy among the OLV cases in the AE group. These surgeries were performed without direct lung invasion. Therefore, further investigation is necessary to assess whether OLV is an independent risk factor for postoperative AEs in non-pulmonary surgeries.

During OLV, the ventilated lung may be exposed to greater stress due to the higher pressure compared to that in two lung ventilation. In the present study, the mean airway pressure in the AE group was 0.9 cmH_2_O higher than that in the non-AE group, and the difference was statistically significant. Low tidal volume and high PEEP management are recommended treatment strategies for ARDS^15,16^. Moreover, high airway pressure can cause ventilator-induced lung injury (VILI)^17^. In 2015, Amato et al. reported that low driving pressure (single ventilation, volume/compliance) is associated with increased survival in patients with ARDS^18^. The probability of postoperative complications has also been reported to increase by 3.4% for every 1 cmH_2_O increase in driving pressure during OLV^19,20^. Considering that the mean airway pressure difference between the AE and non-AE groups in this study was 0.9 cmH_2_O, the effect of driving pressure could be related to AEs.

There was a significant difference in the average mean airway pressure in the AE group in the present study. Driving pressure is often considered an indicator of ventilation management^13^. However, we could not evaluate driving pressure because we could not extract data on tidal volume, respiratory system compliance, and plateau pressure from the anesthesia database. Therefore, it remains unclear whether high mean airway pressure affects the development of postoperative AEs.

Preoperative steroid use is reported to be a predictor of AE following pulmonary resection^6^. The results of the present study suggested that steroid use during general anesthesia may be a predictor of postoperative AE. In the present study, the purpose of steroid use was not limited to prophylaxis for AE (e.g., hydrocortisone for liver resection and methylprednisolone for cardiopulmonary bypass). Moreover, an association has been reported between high average daily steroid use for AE-IPF and in-hospital mortality^21^. Therefore, we should carefully assess perioperative steroid use in patients with ILD.

Several studies have suggested that high F_I_O_2_ levels may damage lung tissue^8–10^. However, in the present study, there was no significant difference in F_I_O_2_ between the AE and non-AE groups. One possible reason for this is that the anesthesiologists in charge who are aware of the risk are expected to maintain inspiratory oxygen levels at the minimum necessary. It is unclear whether AEs occur more frequently when higher oxygen concentrations are administered.

There were no differences between the two groups in the use of volatile or intravenous anesthesia in the present study. We could not determine the anesthesia method that was more favorable in preventing respiratory complications in patients with ILD. More studies are needed to determine whether the choice of anesthesia for patients with ILD is associated with postoperative AEs.

The factors evaluated in the present study are those that anesthesiologists can observe or intervene to treat during the perioperative period. Higher peak pressure might be needed because of a "difficult to ventilate" lung, so that this would be an unmodifiable factor. However, since high airway pressure itself may be the cause of AEs, it is worthwhile to consider lung protective ventilation during surgery. In addition, since OLV may be avoided in pulmonary surgery^22^, it is worthwhile to consider the indication for OLV in patients who are at high risk. Furthermore, the findings of this study can be useful for anesthesiologists in determining perioperative strategies for ILD patients.

### Limitations

Our study has some limitations. First, causality and the effect of unmeasured confounding factors could not be determined, as this was a retrospective study. Second, we were unable to extract the data of some parameters from the anesthesia database. Comparison of the tidal volume, respiratory system compliance, and plateau pressure between patients in the AE and non-AE groups would have facilitated clarification of whether AEs are due to OLV itself or ventilator management. Third, this was a single-center study. Therefore, a study on the management of general anesthesia in patients with ILD at multiple centers is needed.

Although case matching of pulmonary and non-pulmonary surgery requires a large number of cases, we did not perform this study because it was a secondary analysis, and the number of cases was limited. However, we thought it would be worthwhile to examine whether OLV itself contributes to the occurrence of AEs, regardless of whether pulmonary surgery is performed.

We recommend that clinical studies be carried out to determine clinical strategies for better AE management. In addition, a prospective study with a larger number of AEs should be conducted to compare the results.

## Conclusions

Perioperative clinical management was compared between ILD patients with and without postoperative AE when surgery was performed under general anesthesia. OLV was significantly associated with postoperative acute exacerbation of ILD. OLV should be carefully considered in general anesthesia for ILD patients.

### Supplementary Information

Supplementary Information 1.

Supplementary Information 2.

Supplementary Information 3.

Supplementary Information 4.

## Data Availability

The datasets used and analysed during the current study available from the corresponding author on reasonable request.
