# Supplementary material for: Secondary analysis of preoperative predictors for acute postoperative exacerbation in interstitial lung disease
Source: Sci Rep. 2023 Aug 25;13:13955. doi: 10.1038/s41598-023-41152-y (PMC10457368; doi:10.1038/s41598-023-41152-y)
Supplement: Supplementary file 1 — Supplementary Information 1. [file 41598_2023_41152_MOESM1_ESM.docx]

**Supplemental Tables**

**Supplemental table 1: Patients’ surgical site and the procedure with/without OLV in AE group**

| **No.** | **Surgical site** | **OLV(y/n)** |
| --- | --- | --- |
| 1 | Digestive tract | No |
| 2 | Aorta vascular | Yes |
| 3 | Lung | Yes |
| 4 | Lung | Yes |
| 5 | Oto Head Neck oral | No |
| 6 | Neuro | No |
| 7 | Lung | Yes |
| 8 | Lung | Yes |
| 9 | Urology | No |
| 10 | Lung | Yes |
| 11 | Lung | Yes |
| 12 | Heart | No |
| 13 | Lung | Yes |
| 14 | Lung | Yes |
| 15 | Digestive tract | No |
| 16 | Digestive tract | Yes |
| 17 | Lung | Yes |
| 18 | Digestive tract | Yes |
| 19 | Lung | No |
| 20 | Lung | Yes |
| 21 | Lung | Yes |
| 22 | Digestive tract | NO |
| 23 | Lung | Yes |
| 24 | Lung | Yes |
| 25 | Digestive tract | Yes |
| 26 | Lung | Yes |
| 27 | Mediastinum | Yes |
|  |  |  |

**Supplemental table 2: Patients’ surgical site and the procedure with/without OLV in non-AE group**

| **No.** | **Surgical site** | **OLV(y/n)** |
| --- | --- | --- |
| 28 | Aorta vascular | No |
| 29 | Lung | Yes |
| 30 | ObsGyne | No |
| 31 | Hepato-Biliary-Pancreatic-Spleen | No |
| 32 | Orthopedic | No |
| 33 | Orthopedic | No |
| 34 | Lung | Yes |
| 35 | Mediastinum | Yes |
| 36 | Aorta vascular | No |
| 37 | Digestive tract | No |
| 38 | Hepato-Biliary-Pancreatic-Spleen | No |
| 39 | Lung | Yes |
| 40 | Urology | No |
| 41 | Lung | Yes |
| 42 | Lung | Yes |
| 43 | Lung | Yes |
| 44 | Mediastinum | Yes |
| 45 | Aorta vascular | No |
| 46 | Lung | Yes |
| 47 | ObsGyne | No |
| 48 | Heart | No |
| 49 | Orthopedic | No |
| 50 | Digestive tract | No |
| 51 | Lung | Yes |
| 52 | Lung | Yes |
| 53 | Hepato-Biliary-Pancreatic-Spleen | No |
| 54 | Hepato-Biliary-Pancreatic-Spleen | No |
| 55 | Lung | Yes |
| 56 | Hepato-Biliary-Pancreatic-Spleen | No |
| 57 | Digestive tract | No |
| 58 | Digestive tract | No |
| 59 | Hepato-Biliary-Pancreatic-Spleen | No |
| 60 | Lung | Yes |
| 61 | Mediastinum | No |
| 62 | Digestive tract | No |
| 63 | Urology | No |
| 64 | Lung | Yes |
| 65 | Hepato-Biliary-Pancreatic-Spleen | No |
| 66 | Lung | Yes |
| 67 | Oto head neck oral | No |
| 68 | Lung | Yes |
| 69 | Dermatology | No |
| 70 | Dermatology | No |
| 71 | Digestive tract | No |
| 72 | Hepato-Biliary-Pancreatic-Spleen | No |
| 73 | Urology | No |
| 74 | Lung | Yes |
| 75 | Lung | Yes |
| 76 | Urology | No |
| 77 | Digestive tract | Yes |
| 78 | Digestive tract | No |
| 79 | Digestive tract | Yes |
| 80 | Oto head neck oral | No |
| 81 | Urology | No |
| 82 | Lung | Yes |
| 83 | Lung | Yes |
| 84 | Orthopedic | No |
| 85 | Digestive tract | No |
| 86 | Digestive tract | No |
| 87 | Digestive tract | No |
| 88 | Orthopedic | No |
| 89 | Digestive tract | No |
| 90 | Lung | Yes |
| 91 | Orthopedic | No |
| 92 | Orthopedic | No |
| 93 | Digestive tract | No |
| 94 | Lung | Yes |
| 95 | Digestive tract | No |
| 96 | Digestive tract | No |
| 97 | Heart | No |
| 98 | Digestive tract | No |
| 99 | Digestive tract | No |
| 100 | Digestive tract | No |
| 101 | Digestive tract | No |
| 102 | ObsGyne | No |
| 103 | Digestive tract | No |
| 104 | Digestive tract | Yes |
| 105 | Heart | No |
| 106 | Orthopedic | No |
| 107 | Lung | Yes |
| 108 | Digestive tract | No |
| 109 | Digestive tract | No |
| 110 | Othrs | No |
| 111 | Neuro | No |
| 112 | Urology | No |
| 113 | Digestive_tract | No |
| 114 | Heart | No |
| 115 | Hepato-Biliary-Pancreatic-Spleen | No |
| 116 | Aorta vascular | No |
| 117 | Digestive tract | No |
| 118 | Orthopedic | No |
| 119 | Oto head neck oral | No |
| 120 | Mediastinum | Yes |
| 121 | Mediastinum | No |
| 122 | Hepato-Biliary-Pancreatic-Spleen | No |
| 123 | Digestive tract | No |
| 124 | Orthopedic | No |
| 125 | Lung | Yes |
| 126 | Lung | Yes |
| 127 | Oto head neck oral | No |
| 128 | Aorta vascular | No |
| 129 | Lung | Yes |
| 130 | Lung | Yes |
| 131 | Digestive tract | No |
| 132 | Digestive tract | No |
| 133 | Digestive tract | No |
| 134 | Digestive tract | No |
| 135 | Breast | No |
