# Supplementary material for: Secondary analysis of preoperative predictors for acute postoperative exacerbation in interstitial lung disease
Source: Sci Rep. 2023 Aug 25;13:13955. doi: 10.1038/s41598-023-41152-y (PMC10457368; doi:10.1038/s41598-023-41152-y)
Supplement: Supplementary file 2 — Supplementary Information 2. [file 41598_2023_41152_MOESM2_ESM.docx]

**Supplemental Figure legend**

**Supplemental figure S1:** **The percentage of patients’ surgical sites in AE group**

**Supplemental figure S2:** **The percentage of patients’ surgical sites in non-AE group**
