# Supplementary figures and images for: Secondary analysis of preoperative predictors for acute postoperative exacerbation in interstitial lung disease
Source: Sci Rep. 2023 Aug 25;13:13955. doi: 10.1038/s41598-023-41152-y (PMC10457368; doi:10.1038/s41598-023-41152-y)

**Supplemental figure S1****
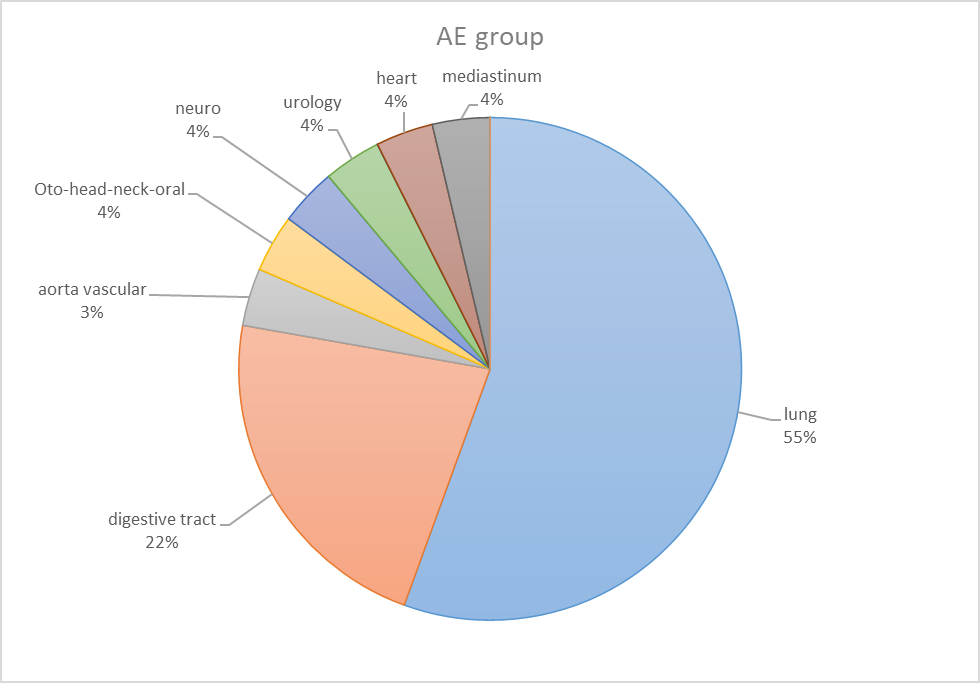
**

Supplement: Supplementary file 3 — Supplementary Information 3. [file 41598_2023_41152_MOESM3_ESM.docx]

**Supplemental figure S2**


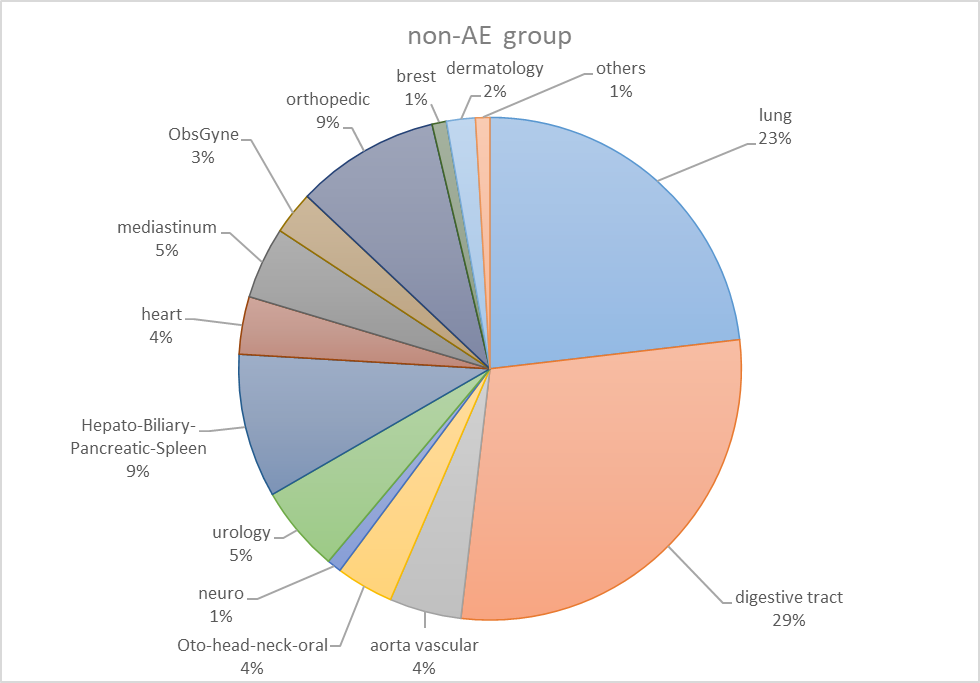

Supplement: Supplementary file 4 — Supplementary Information 4. [file 41598_2023_41152_MOESM4_ESM.docx]
